# Non-contiguous finished genome sequence and description of *Alistipes ihumii* sp. nov.

**DOI:** 10.4056/sigs.4698398

**Published:** 2014-02-15

**Authors:** Anne Pfleiderer, Ajay Kumar Mishra, Jean-Christophe Lagier, Catherine Robert, Aurelia Caputo, Didier Raoult, Pierre-Edouard Fournier

**Affiliations:** 1 Aix-Marseille Université, URMITE, UM63, Faculté de médecine, Marseille, France; 2 King Fahd Medical Research Center, King Abdul Aziz University, Jeddah, Saudi Arabia

**Keywords:** *Alistipes ihumii*, genome, culturomics, taxono-genomics

## Abstract

*Alistipes ihumii* strain AP11^T^ sp. nov. is the type strain of *A. ihumii* sp. nov., a new species within the genus *Alistipes*. This strain, whose genome is described here, was isolated from the fecal flora of a 21-year-old French Caucasian female, suffering from a severe restrictive form of anorexia nervosa since the age of 12 years. *A. ihumii* is a Gram-negative anaerobic bacillus. Here we describe the features of this organism, together with the complete genome sequence and annotation. The 2,753,264 bp long genome (one chromosome but no plasmid) contains 2,254 protein-coding and 47 RNA genes, including 3 rRNA genes.

## Introduction

*Alistipes ihumii* strain AP11^T^ (= CSUR P204 = DSM 26107) is the type strain of *A. ihumii* sp. nov. This bacterium is a Gram-negative, non-spore-forming, anaerobic and non-motile bacillus that was isolated from the stool of a 21-year-old French female suffering from anorexia nervosa, and is part of a “culturomics” study aiming at cultivating individually all species within human feces [1-3].

Prokaryotic taxonomy is episodically confronted with the advancement of methodological and conceptual innovations. The current classification methodology for prokaryotes is known as polyphasic taxonomy, and relies on a combination of phenotypic and genotypic characteristics [4]. The number of completely sequenced genomes is geometrically increasing with time, concurrently with the decrease in cost of such techniques. To date, more than 6,000 bacterial genomes have been published and approximately 25,000 genome sequencing projects have been announced [5]. We recently proposed to integrate genomic information in the taxonomic framework for the description of new bacterial species [6-27].

The genus *Alistipes* (Rautio *et al*. 2003) was created in 2003 [28] and is composed of strictly anaerobic Gram-negative rods that resemble the *Bacteroides fragilis* group in that most species are bile-resistant and indole-positive [29]. This genus is currently comprised of five species with validly published names, including *A. finegoldii*, *A. putredinis* [28], *A. indistinctus* [30], *A. onderdonkii* and *A. shahii* [31], to which we added three proposed new species, *A. senegalensis* [8], *A. timonensis* [9] and *A. obesi* [22].

Here we present a summary classification and a set of features for a new *Alistipes* species, *A. ihumii* sp. nov. strain AP11^T^ (= CSUR P204 = DSM 26107), together with the description of the complete genomic sequence and its annotation.

## Classification and features

A stool sample was collected from a 21-year-old French Caucasian female suffering from severe restrictive form of anorexia nervosa since the age of 12 years. At the time of sample collection, she was hospitalized in our hospital for recent aggravation of her medical condition (BMI: 10.4 kg/m^2^). The patient gave an informed and signed consent. Both this study and the assent procedure were approved by the Ethics Committee of the Institut Fédératif de Recherche IFR48, Faculty of Medicine, Marseille, France under reference 09-022. Ten other potentially new bacterial species were isolated from this patient’s stool, all of which are currently being described. Microbial culturomics also enabled the isolation of several other new bacterial species from other stool specimens [6-27]. The fecal specimen was stored at -80°C immediately after collection. Strain AP11^T^ was isolated in November 2011 after 2 days of inoculation in anaerobic blood culture bottle with the addition of 5mL of thioglycolate and further inoculation on Columbia agar (BioMerieux, Marcy l’Etoile, France).

**Figure 1:**
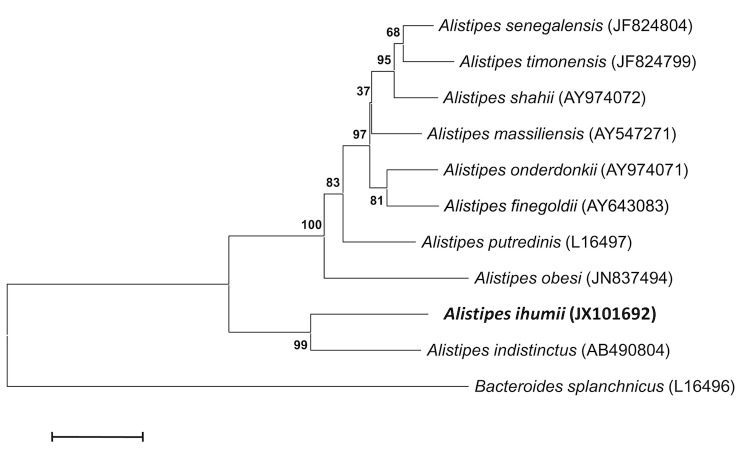
Phylogenetic tree highlighting the position of *Alistipes ihumii* strain AP11^T^ relative to other type strains within the genus *Alistipes*. GenBank accession numbers are indicated in parentheses. Sequences were aligned using CLUSTALW, and phylogenetic inferences obtained using the maximum-likelihood method within the MEGA software. Numbers at the nodes are percentages of bootstrap values obtained by repeating the analysis 500 times to generate a majority consensus tree. *Bacteroides splanchnicus* was used as the outgroup. The scale bar represents a 2% nucleotide sequence divergence.

This strain exhibited a 95% 16S rRNA sequence similarity with *A. indistinctus* [30], the phylogenetically closest *Alistipes* species with a validly published name (Table 1, Figure 1), and 92% with *A. onderdonkii* [28] and *A. putredinis* [31]. This value was in the range of 16S rRNA sequence identities among species within the genus *Alistipes* that range from 90 to 95%, and lower than the 98.7% 16S rRNA gene sequence threshold recommended by Stackebrandt and Ebers to delineate a new species without carrying out DNA-DNA hybridization [41].

**Table 1:** Classification and general features of *Alistipes ihumii* strain AP11^T^ according to the MIGS recommendations [32]

**MIGS ID**	**Property**	**Term**	**Evidence code^a^**
	Current classification	Domain *Bacteria* Phylum *Bacteroidetes* Class *Bacteroidia* Order *Bacteroidales* Family *Rikenellaceae* Genus *Alistipes* Species *Alistipes ihumii* Type strain AP11^T^	TAS [33] TAS [34,35] TAS [34,36] TAS [34,37] TAS [34,38] TAS [28,39] IDA IDA
	Gram stain	Negative	IDA
	Cell shape	Rod	IDA
	Motility	nonmotile	IDA
	Sporulation	nonsporulating	IDA
	Temperature range	mesophile	IDA
	Optimum temperature	37°C	IDA
MIGS-6.3	Salinity	unknown	IDA
MIGS-22	Oxygen requirement	anaerobic	IDA
	Carbon source	unknown	
	Energy source	unknown	
MIGS-6	Habitat	human gut	IDA
MIGS-15	Biotic relationship	free living	IDA
MIGS-14	Pathogenicity Biosafety level Isolation	unknown 2 human feces	
MIGS-4	Geographic location	France	IDA
MIGS-5	Sample collection time	November 2011	IDA
MIGS-4.1	Latitude & Longitude	43.296482 & 5.36978	IDA
MIGS-4.3	Depth	surface	IDA
MIGS-4.4	Altitude	0 m above sea level	IDA

Evidence codes - IDA: Inferred from Direct Assay; TAS: Traceable Author Statement (i.e., a direct report exists in the literature); NAS: Non-traceable Author Statement (i.e., not directly observed for the living, isolated sample, but based on a generally accepted property for the species, or anecdotal evidence). These evidence codes are from the Gene Ontology project [40]. If the evidence is IDA, then the property was directly observed for a live isolate by one of the authors or an expert mentioned in the acknowledgements.

**Figure 2:**
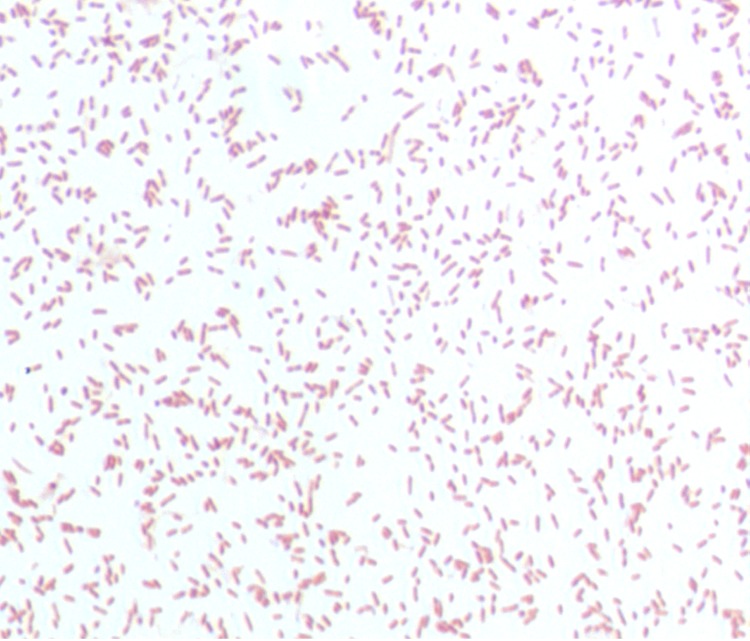
Gram stain of *A. ihumii* strain AP11^T^

**Figure 3:**
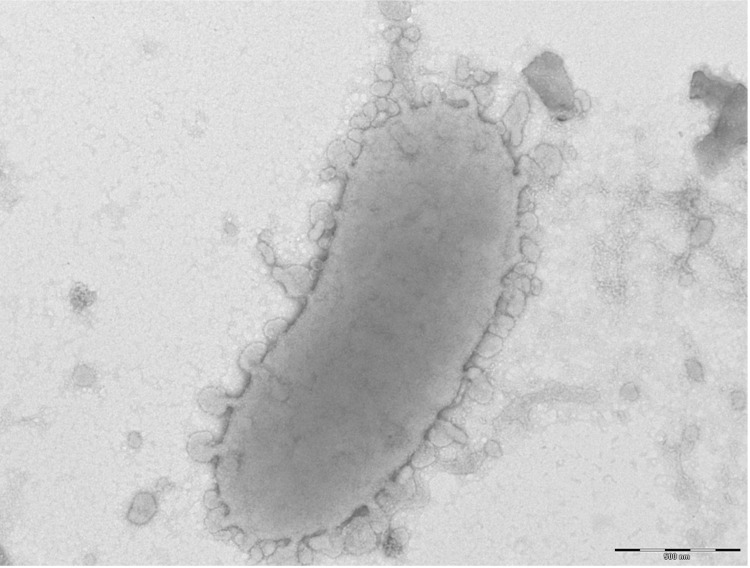
Transmission electron microscopy of *A. ihumii* strain AP11^T^, using a Morgani 268D (Philips) at an operating voltage of 60kV. The scale bar represents 500 nm.

Different growth temperatures (25, 30, 37, 45°C) were tested. Growth was observed between 25 and 45°C, with optimal growth at 37°C after 24 hours of inoculation. Colonies were about 0.2 mm in diameter, transparent, and exhibited a ß-hemolytic activity on blood-enriched Columbia agar. Growth of the strain was tested on 5% sheep blood agar, under anaerobic and microaerophilic conditions using the GENbag anaer and GENbag microaer systems, respectively (BioMerieux), and under aerobic conditions with or without 5% CO_2_. Optimal growth of this strain was obtained anaerobically, weak growth was observed under microaerophilic conditions, and no growth was observed under aerobic atmosphere. The motility test was negative. Cells grown on agar are Gram-negative rods (Figure 2) and have mean diameter and length of 0.72 and 1.69 µm, respectively, as determined using electron microscopy (Figure 3).

Strain AP11^T^ exhibited oxidase but no catalase activities. Using API 50CH (BioMérieux), we observed that strain AP11^T^ was asaccharolytic. Using API 32A (BioMérieux), positive reactions were obtained for α-glucosidase, β-glucosidase, N-acetyl-β-glucosaminidase, mannose and raffinose fermentation, alkaline phosphatase, leucyl glycine arylamidase, alanine arylamidase, and glutamyl glutamic acid arylamidase. Weak reactions were observed for α-galactosidase and glutamic acid decarboxilase. Negative reactions were obtained for urease, arginine dihydrolase, β-galactosidase, 6 phospho-β-galactosidase , α-arabinosidase, β-glucuronidase, α-fucosidase, nitrate reduction, indole production, arginine arylamidase, proline arylamidase, phenylalanine arylamidase, leucine arylamidase, pyroglutamic acid arylamidase, tyrosine arylamidase, glycine arylamidase, histidine arylamidase, and serine arylamidase. *A. ihumii* is susceptible to amoxicillin, imipenem, and clindamycin, but resistant to vancomycin. When compared with representative species from the genus *Alistipes*, strain AP11^T^ exhibited the phenotypic differences detailed in Table 2.

**Table 2:** Differential characteristics of *Alistipes* strains^†^

**Properties**	*A. ihumii*	*A. senegalensis*	*A.* *timonensis*	*A.* *putredinis*	*A.* *indistinctus*	*A.* *shahii*	*A.* *obesi*	*A.* *finegoldii*
Cell diameter (µm)	0.72	0.56	0.62	0.40	0.60	0.15	0.44-0.76	0.8-2.0
Oxygen requirement	anaerobic	anaerobic	anaerobic	anaerobic	anaerobic	anaerobic	anaerobic	anaerobic
Pigment production	–	+	+	–	+	+	+	+
Gram stain	–	–	–	–	–	–	–	–
Salt requirement	na	+	–	–	–	–	–	na
Motility	–	–	–	–	–	–	+	–
Endospore formation	–	–	–	–	–	–	na	–
** **								
**Production of**								
Alkaline phosphatase	na	na	na	+	w	+	+	+
Catalase	–	+	+	+	+	–	+	–
Oxidase	+	–	–	–	–	–	–	na
Nitrate reductase	–	na	na	–	–	–	–	–
Urease	–	na	na	–	–	+	–	na
β-galactosidase	–	w	+	–	–	+	+	+
N-acetyl-glucosamine	+	na	W	–	+	+	+	+
Indole	–	w	W	+	–	+	–	+
** **								
**Activity for**								
Leucyl glycine arylamidase	+	+	+	+	–	+	+	–
Glutamic acid decarboxylase	w	na	+	+	–	–	–	na
Glycine arylamidase	–	+	+	na	–	–	–	na
Chymotrypsin	na	na	na	–	–	–	na	na
** **								
**Acid from**								
L-Arabinose	na	na	na	–	+	na	na	na
Raffinose	+	na	–	–	+	+	–	na
Mannose	+	+	–	–	+	+	–	na
Mannitol	na	na	na	na	+	na	na	na
Sucrose	na	na	na	–	+	+	na	na
D-glucose	na	na	na	–	+	+	na	na
D-fructose	na	na	na	–	+	+	na	na
D-maltose	na	na	na	–	+	+	na	na
D-lactose	na	na	na	–	+	+	na	na
** **								
**Hydrolysis of gelatin**	na	na	na	+	+	–	na	+
G+C content (mol%)	57.90	58.40	58.82	55.3	55.2	57.20	58.60	56.65
Habitat	human gut	human gut	human gut	appendix of children	human gut	human gut	human gut	human appendix tissue

na = data not available; w = weak

^†^*Alistipes ihumii* strain AP11^T^, *A. senegalensis* strain JC50^T^, *A. timonensis* strain JC136^T^, *A. putredinis* strain ATCC29800 ^T^, *A. indistinctus* strain YIT12060^T^, *A. shahii* strain WAL 8301^T^, *A. obesi* strain ph8^T^ and *A. finegoldii* AHN2437^T^

**Figure 4:**
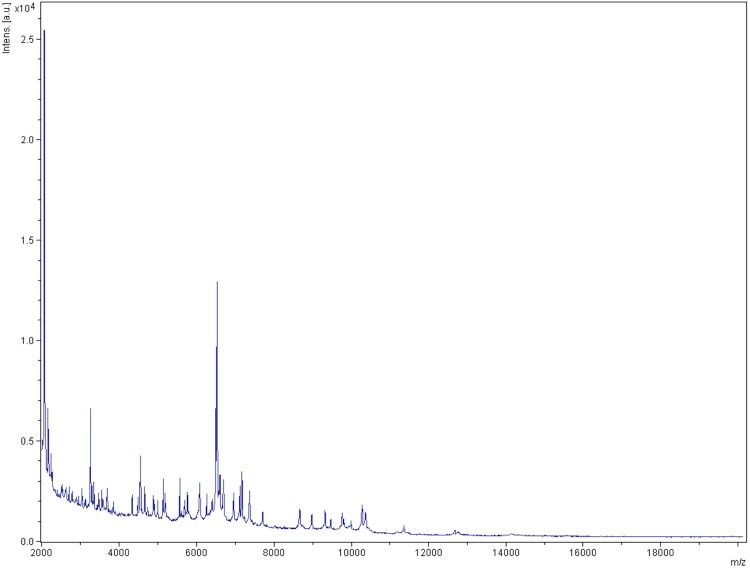
Reference mass spectrum from *A. ihumii* strain AP11^T^. Spectra from 12 individual colonies were compared and a reference spectrum was generated.

**Figure 5:**
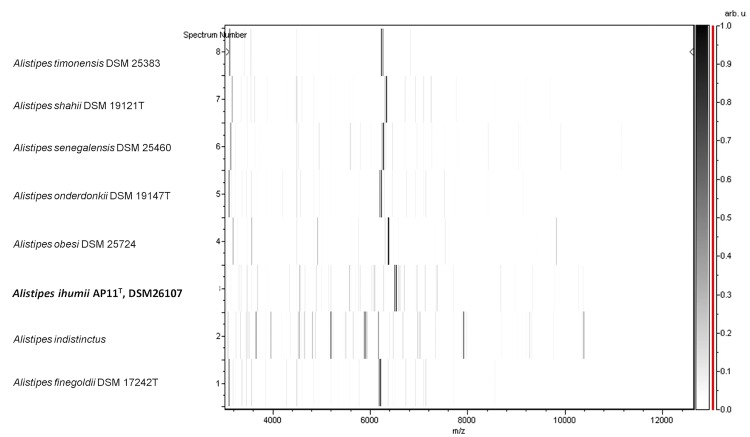
Gel view comparing spectra from *Alistipes ihumii* strain AP11^T^ and other members of the genus *Alistipes* (*A. obesi, A. timonensis*, *A. senegalensis*, *A. shahii*, *A. onderdonkii* and *A. finegoldii*). The Gel View displays the raw spectra of all loaded spectrum files arranged in a pseudo-gel like look, with each peak displayed as a band or bar. The peak intensity is reflected by the intensity of the gray color. The right y-axis shows the relationship between the shades of gray and the peak intensity in arbitrary units. The x-axis records the m/z value. The left y-axis displays the running spectrum number originating from subsequent spectra loading.

Matrix-assisted laser-desorption/ionization time-of-flight (MALDI-TOF) MS protein analysis was carried out as previously described [42] using a Microflex spectrometer (Bruker Daltonics, Leipzig, Germany). Twelve individual colonies were deposited on a MTP 384 MALDI-TOF target plate (Bruker). The twelve AP11^T^ spectra were imported into the MALDI BioTyper software (version 2.0, Bruker) and analyzed by standard pattern matching (with default parameter settings) against the main spectra of 4,706 bacteria, including spectra from *A. finegoldii*, *A. onderdonkii*, *A. shahii*, *A. senegalensis*, *A. obesi* and *A. timonensis*, used as reference data in the BioTyper database. The output score enabled the presumptive identification and discrimination of the tested species from those in the database: a score > 2 with a validated species identifies a strain at the species level; and a score < 1.7 indicates a species-level match was not made. For strain AP11^T^, no significant score was obtained, suggesting that our isolate was not a member of any known species (Figures 4 and 5). We added the spectrum from strain AP11^T^ to our database.

## Genome sequencing information

### Genome project history

The organism was selected for sequencing on the basis of its phylogenetic position and 16S rRNA similarity to other members of the *Alistipes* genus, and is part of a “culturomics” study of the human digestive flora aiming at isolating all bacterial species within human feces. It was the eighth sequenced genome from an *Alistipes* species and the first from *Alistipes ihumii* sp. nov. A summary of the project information is shown in Table 3. The Genbank accession number is CAPH00000000 and consists of 60 contigs. Table 3 shows the project information and its association with MIGS version 2.0 compliance [43].

**Table 3:** Project information

**MIGS ID**	**Property**	**Term**
MIGS-31	Finishing quality	High-quality draft
MIGS-28	Libraries used	One 454 paired end 3-kb library
MIGS-29	Sequencing platforms	454 GS FLX Titanium
MIGS-31.2	Fold coverage	35×
MIGS-30	Assemblers	Newbler version 2.5.3
MIGS-32	Gene calling method	Prodigal
	Genbank ID	CAPH00000000
	Genbank Date of Release	November 28, 2012
	Gold ID	Gi20720
MIGS-13	Project relevance	Study of the human gut microbiome

### Growth conditions and DNA isolation

*A. ihumii* sp. nov. strain AP11^T^, (= CSURP204 = DSM 26107), was grown aerobically on 5% sheep blood agar medium at 37°C. Five Petri dishes were spread and resuspended in 3x100µl of G2 buffer (EZ1 DNA Tissue kit, Qiagen). A first mechanical lysis was performed by glass powder on the Fastprep-24 device (Sample Preparation system, MP Biomedicals, USA) for 2×20 seconds. DNA was treated with 2.5 µg/µL of lysozyme (30 minutes at 37°C) and extracted using the BioRobot EZ 1 Advanced XL (Qiagen). The DNA was concentrated and purified on a Qiamp kit (Qiagen). The yield and the concentration of DNA was 70.7 ng/µl as measured by using Quant-it Picogreen kit (Invitrogen) on the Genios Tecan fluorometer.

### Genome sequencing and assembly

A 3kb paired-end sequencing strategy (Roche, Meylan, France) was used. DNA (5 µg) was mechanically fragmented for the paired-end sequencing, using a Covaris device (Covaris Inc., Woburn, MA,USA) with an enrichment size of 3-4 kb. The DNA fragmentation was visualized through an Agilent 2100 BioAnalyzer on a DNA Labchip 7500 which yielded an optimal size of 2.3 kb. The library was constructed using the 454 GS FLX Titanium paired-end rapid library protocol. Circularization and nebulization were performed which generated a pattern of optimal size of 457 bp. PCR amplification was performed for 17 cycles followed by double size selection. The single-stranded paired-end library was quantified using a Quant-it Ribogreen Kit (Invitrogen) and the Genios Tecan fluorometer. The library concentration equivalence was calculated as 1.94× 10^10^ molecules/µL. The library was stored at -20°C until further use.

The paired-end library was clonally amplified with 0.5 and 1 cpb in 2 emPCR reactions with the GS Titanium SV emPCR Kit (Lib-L) v2 (Roche). The yield of the shotgun emPCR reactions was 6.24 and 16.24% respectively for the two kinds of paired- end emPCR reactions according to the quality expected (range of 5 to 20%) from the Roche procedure. Two libraries were loaded on the GS Titanium PicoTiterPlates (PTP Kit 70x75, Roche) and pyrosequenced with the GS Titanium Sequencing Kit XLR70 and the GS FLX Titanium sequencer (Roche). The run was performed overnight and then analyzed on the cluster through the gsRunBrowser and Newbler assembler (Roche). A total of 260,838 passed filter wells were obtained and generated 96.3 Mb with an average length of 369 bp. The passed filter sequences were assembled using Newbler with 90% identity and 40 bp as overlap. The final assembly identified 9 scaffolds and 60 contigs (> 1,500 bp) and generated a genome size of 2.75 Mb which corresponds to a coverage of 35× genome equivalent.

### Genome annotation

Open Reading Frames (ORFs) were predicted using Prodigal [44] with default parameters but the predicted ORFs were excluded if they were spanning a sequencing gap region. The predicted bacterial protein sequences were searched against the GenBank database [45] and the Clusters of Orthologous Groups (COG) databases using BLASTP. The tRNAScan-SE tool [46] was used to find tRNA genes, whereas ribosomal RNAs were found by using RNAmmer [47] and BLASTn against the GenBank database. Lipoprotein signal peptides and numbers of transmembrane helices were predicted using SignalP [48] and TMHMM [49] respectively. ORFans were identified if their BLASTP *E*-value was lower than 1e^-03^ for alignment length greater than 80 amino acids. If alignment lengths were smaller than 80 amino acids, we used an *E*-value of 1e^-05^. Such parameter thresholds have already been used in previous works to define ORFans.

Orthologous gene sets composed of one gene from *A. ihumii* compared to each of *A. obesi* strain ph8^T^ (GenBank accession number CAHA00000000), *A. finegoldii* strain AHN 2437 (CP003274), *A. indistinctus* strain YIT 12060 (ADLD00000000), *A. putredinis* strain DSM 17216 (ABFK00000000), *A. senegalensis* strain JC50^T^ (CAHI00000000), *A. shahii* strain WAL 8301 (FP929032), and *A. timonensis* strain JC136^T^ (CAEG00000000) were identified using the Proteinortho software (version 1.4) [50] using a 30% protein identity and an *E*-value of 1e^-05^. The average percentage of nucleotide sequence identity of each orthologous set was determined using the Needleman-Wunsch algorithm global alignment technique. Artemis [51] was used for data management and DNA Plotter [52] was used for visualization of genomic features. The Mauve alignment tool was used for multiple genomic sequence alignment and visualization [53].

## Genome properties

**Figure 6:**
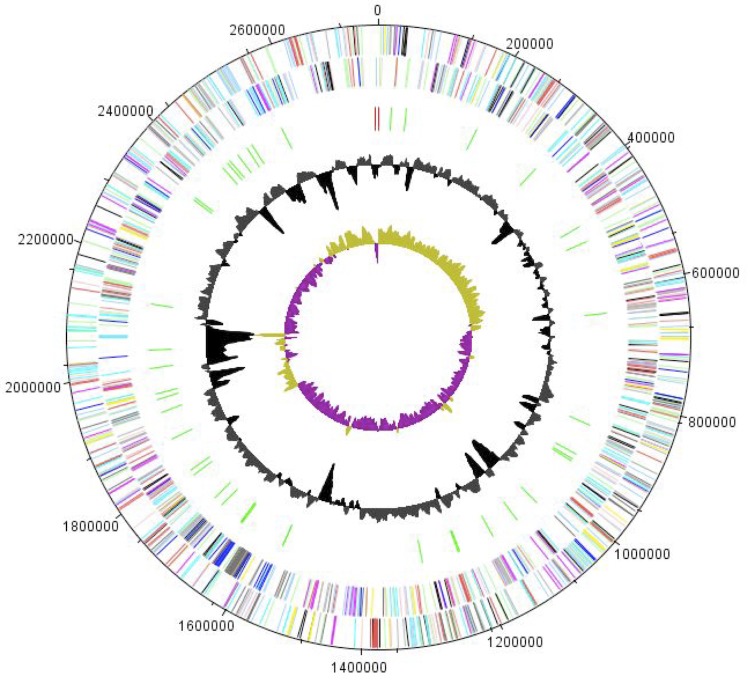
Graphical circular map of the chromosome. From the outside in, the outer two circles show open reading frames oriented in the forward and reverse (colored by COG categories) directions, respectively. The third circle marks the rRNA gene operon (red) and tRNA genes (green). The fourth circle shows the G+C% content plot. The inner-most circle shows GC skew, purple and olive indicating negative and positive values, respectively.

The genome of *A. ihumii* strain AP11^T^ is 2,753,264 bp long (1 chromosome, but no plasmid) with a 57.90% G + C content (Figure 6 and Table 4). Of the 2,301 predicted genes, 2,254 were protein-coding genes, and 47 were RNAs. One rRNA operon (one 16S rRNA, one 23S rRNA and one 5S rRNA) and 44 predicted tRNA genes were identified in the genome. A total of 1,465 genes (63.66%) were assigned a putative function. Two hundred thirty-seven genes were identified as ORFans (10.29%). The remaining genes were annotated as hypothetical proteins. The properties and the statistics of the genome are summarized in Tables 4 and 5. The distribution of genes into COGs functional categories is presented in Table 5.

**Table 4:** Nucleotide content and gene count levels of the genome

**Attribute**	Value	% of total^a^
Genome size (bp)	2,753,264	
DNA coding region (bp)	2,320,878	84.29
DNA G+C content (bp)	1,594,140	57.90
Number of replicons	1	
Extrachromosomal elements	0	
Total genes	2,301	100
RNA genes	47	2.04
rRNA operons	1	
Protein-coding genes	2,254	97.95
Genes with function prediction	1,540	66.92
Genes assigned to COGs	1,465	63.66
Protein coding genes assigned Pfam domains	1,834	79.70
Genes with peptide signals	296	12.86
Genes with transmembrane helices	457	19.86
CRISPR repeats	1	

^a^ The total is based on either the size of the genome in base pairs or the total number of protein coding genes in the annotated genome.

**Table 5:** Number of genes associated with the 25 general COG functional categories

**Code**	**Value**	**%age**^a^	**Description**
J	143	6.34	Translation
A	0	0	RNA processing and modification
K	88	3.90	Transcription
L	113	5.01	Replication, recombination and repair
B	0	0	Chromatin structure and dynamics
D	19	0.84	Cell cycle control, mitosis and meiosis
Y	0	0	Nuclear structure
V	28	1.24	Defense mechanisms
T	38	1.69	Signal transduction mechanisms
M	161	7.14	Cell wall/membrane biogenesis
N	6	0.27	Cell motility
Z	0	0	Cytoskeleton
W	0	0	Extracellular structures
U	32	142	Intracellular trafficking and secretion
O	60	2.66	Posttranslational modification, protein turnover, chaperones
C	111	4.92	Energy production and conversion
G	106	4.70	Carbohydrate transport and metabolism
E	131	5.81	Amino acid transport and metabolism
F	52	2.31	Nucleotide transport and metabolism
H	75	3.33	Coenzyme transport and metabolism
I	49	2.17	Lipid transport and metabolism
P	72	3.19	Inorganic ion transport and metabolism
Q	21	0.93	Secondary metabolites biosynthesis, transport and catabolism
R	235	10.43	General function prediction only
S	91	4.04	Function unknown
-	790	35.05	Not in COGs

^a^The total is based on the total number of protein coding genes in the annotated genome.

## Genome comparison with other *Alistipes* species

Here, we compared the genome of *A. ihumii* strain AP11^T^ to those of *A. obesi* strain ph8^T^ (GenBank accession number CAHA00000000), *A. finegoldii* strain AHN 2437 (CP003274), *A. indistinctus* strain YIT 12060 (ADLD00000000), *A. putredinis* strain DSM 17216 (ABFK00000000), *A. senegalensis* strain JC50^T^ (CAHI00000000), *A. shahii* strain WAL 8301 (FP929032), and *A. timonensis* strain JC136^T^ (CAEG00000000). The draft genome of *A. ihumii* is larger than that of *A. putredinis* (2.75 and 2.55 Mb, respectively) but smaller than those of *A. indistinctus*, *A. obesi*, *A. timonensis*, *A. finegoldii*, *A. shahii* and *A. senegalensis* (2.85, 3.16, 3.49, 3.73, 3.76, and 4.01 Mb, respectively). The G+C content of *A. ihumii* is comparable to that of *A. shahii* (57.90 and 57.60%, respectively), lower than those of *A. timonensis* and *A. senegalensis* (58.8 and 58.4%, respectively) and higher than those of *A. putredinis,* *A. indistinctus* and *A. finegoldii* (53.30, 54.80 and 56.60%, respectively). *A. ihumii* has a smaller gene content than those of *A. putredinis*, *A. indistinctus*, *A. obesi*, *A. timonensis*, *A. shahii*, *A. senegalensis,* and *A. finegoldii* (2,301, 2,335, 2,342, 2,619, 2,709, 3,132, 3,161, and 3,231, respectively). The ratio of genes per MB of *A. ihumii* is higher than those of *A. timonensis*, *A. senegalensis*, *A. indistinctus,* and *A. obesi* (836, 776, 788, 821, and 828, respectively), comparable to that of *A. shahii* (833) and smaller than those of *A. finegoldii* and *A. putredinis* (866 and 915, respectively).

The average genomic nucleotide sequence identity between *A. ihumii* and other *Alistipes* species ranged from 70.23 to 74.37%, whereas values ranged from 69.70 to 90.98% among other *Alistipes* species (Table 6).

**Table 6:** Numbers of orthologous proteins shared between genomes

	AIH	ASE	AT	AS	AF	AP	AO	AIN
*A. ihumii*	**2,254**	1,190	1,164	958	1,150	1,055	1,130	1,147
*A. senegalensis*	71.16	**3,161**	1,764	1,739	1,660	1,277	1,405	1,218
*A.timonensis*	70.90	90.98	**2,709**	1,650	1,585	1,238	1,377	1,210
*A.shahii*	71.19	86.33	80.03	**3,132**	1,674	1,270	1,166	1,155
*A.finegoldii*	71.62	82.04	81.14	82.90	**3,231**	1,303	1,385	1,202
*A.putrenidis*	70.23	75.32	75.21	75.50	76.23	**2,335**	1,182	1,038
*A.onderdonkii*	71.26	76.42	76.23	77.06	76.31	74.45	**2,619**	1,137
*A.indistinctus*	74.37	70.02	70.05	70.00	69.91	69.70	69.91	**2,342**

Upper right triangle- numbers of orthologous proteins shared between genomes

Lower left triangle- average percentage of nucleotide identity between orthologous gene sets shared between genomes

Bold- numbers of proteins per genome

AIH- *A. ihumii*, ASE- *A. senegalensis*, AT- *A. timonensis*, AS- *A. shahii,* AF- *A. finegoldii*, AP- *A. putredinis*, AO- *A. obesi*, AIN- *A. indistinctus*

**Figure 7:**
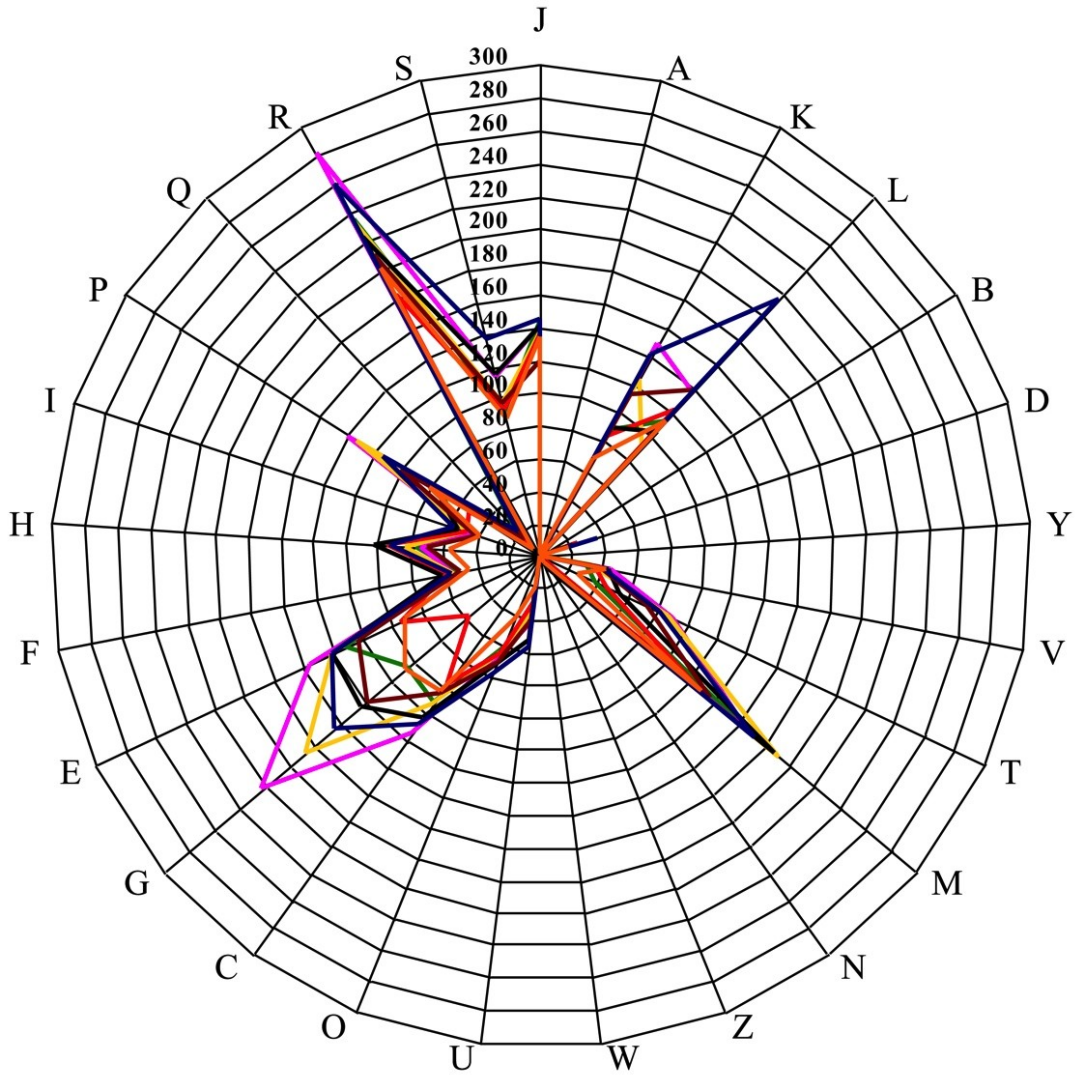
Distribution of functional classes of predicted genes in *Alistipes ihumii* (colored in green), *A. senegalensis* (pink), *A. timonensis* (yellow), *A. shahii* (brown), *A. finegoldii* (blue), *A. putredinis* (red), *A. obesi* (orange) and *A. indistinctus* (black) chromosomes according to the clusters of orthologous groups of proteins.

However, the distribution of genes into COG categories was not entirely similar in all eight compared genomes (Figure 7).

## Conclusion

On the basis of phenotypic, phylogenetic and genomic analyses, we formally propose the creation of *Alistipes ihumii* sp. nov. that contains strain AP11^T^. This bacterial strain has been isolated from the fecal flora of a patient suffering from anorexia nervosa living in Marseille, France. Several other new bacterial species were also cultivated from this patient as well as fecal samples from other patients using microbial culturomics [6-27], thus suggesting that the human fecal flora from human remains partially unknown.

### Description of *Alistipes ihumii* sp. nov.

*Alistipes ihumii* (i.hum.i’i. N.L. gen. n. ihumii, based on the acronym IHUMI, the Institut Hospitalo-Universitaire Méditerranée-Infection, where the type strain was isolated).

Colonies are 0.2 mm in diameter and are translucent on blood-enriched Columbia agar. Cells are rod-shaped with a mean diameter of 0.72 µm and a mean length of 1.69 µm. Optimal growth is achieved anaerobically. No growth is obtained aerobically but weak growth is observed in microaerophilic conditions. Growth occurs between 25°C and 45°C, with an optimal growth observed at 37°C.

Cells stain Gram-negative, are non motile and are asaccharolytic. Activities present are α-glucosidase, β-glucosidase, N-acetyl-β-glucosaminidase, mannose and rafinnose fermentation, alkaline phosphatase, leucyl glycine arylamidase, alanine arylamidase, and glutamyl glutamic acid arylamidase. Cells are negative for urease, arginine dihydrolase, β-galactosidase, 6-phospho-β-galactosidase, α-arabinosidase, β-glucuronidase, α-fucosidase, nitrate reduction, indole production, arginine arylamidase, proline arylamidase, phenylalanine arylamidase, leucine arylamidase, pyroglutamic acid arylamidase, tyrosine arylamidase, glycine arylamidase, histidine arylamidase, and serine arylamidase. Cells are susceptible to amoxicillin, imipenem, and clindamycin, but resistant to vancomycin. The G+C content of the genome is 57.90%. The 16S rRNA and genome sequences are deposited in Genbank under accession numbers JX101692 and CAPH00000000, respectively.

The type strain AP11^T^ (= CSUR P204 = DSM 26107) was isolated from the fecal flora of a 21-year-old French Caucasian female suffering from severe anorexia nervosa.
